# Gene-Splitting Technology: A Novel Approach for the Containment of Transgene Flow in *Nicotiana tabacum*

**DOI:** 10.1371/journal.pone.0099651

**Published:** 2014-06-10

**Authors:** Xu-Jing Wang, Xi Jin, Bao-Qing Dun, Ning Kong, Shi-Rong Jia, Qiao-Ling Tang, Zhi-Xing Wang

**Affiliations:** 1 Biotechnology Research Institute, Chinese Academy of Agricultural Sciences, Beijing, China; 2 National Key Facility for Crop Gene Resources and Genetic Improvement, Institute of Crop Sciences, Chinese Academy of Agricultural Sciences, Beijing, China; Instituto de Biotecnología, Universidad Nacional Autónoma de México, Mexico

## Abstract

The potential impact of transgene escape on the environment and food safety is a major concern to the scientists and public. This work aimed to assess the effect of intein-mediated gene splitting on containment of transgene flow. Two fusion genes, *EPSPSn-In* and *Ic-EPSPSc*, were constructed and integrated into *N. tabacum*, using *Agrobacterium tumefaciens*-mediated transformation. *EPSPSn-In* encodes the first 295 aa of the herbicide resistance gene 5-enolpyruvyl shikimate-3-phosphate synthase (EPSPS) fused with the first 123 aa of the Ssp DnaE intein (In), whereas *Ic-EPSPSc* encodes the 36 C-terminal aa of the Ssp DnaE intein (Ic) fused to the rest of EPSPS C terminus peptide sequences. Both *EPSPSn-In* and *Ic-EPSPSc* constructs were introduced into the same *N. tabacum* genome by genetic crossing. Hybrids displayed resistance to the herbicide N-(phosphonomethyl)-glycine (glyphosate). Western blot analysis of protein extracts from hybrid plants identified full-length EPSPS. Furthermore, all hybrid seeds germinated and grew normally on glyphosate selective medium. The 6-8 leaf hybrid plants showed tolerance of 2000 ppm glyphosate in field spraying. These results indicated that functional EPSPS protein was reassembled *in vivo* by intein-mediated trans-splicing in 100% of plants. In order to evaluate the effect of the gene splitting technique for containment of transgene flow, backcrossing experiments were carried out between hybrids, in which the foreign genes *EPSPSn-In* and *Ic-EPSPSc* were inserted into different chromosomes, and non-transgenic plants NC89. Among the 2812 backcrossing progeny, about 25% (664 plantlets) displayed glyphosate resistance. These data indicated that transgene flow could be reduced by 75%. Overall, our findings provide a new and highly effective approach for biological containment of transgene flow.

## Introduction

Along with the rapid development and commercialization of genetic modified crops worldwide [1], the potential impact of transgene flow mediated by pollen dispersal on the environment and food safety has become a major concern to the scientific community as well as the public. To date, spatial or temporal isolation is generally applied to control transgene flow, but these methods generally show limited efficacy. To further minimize and hopefully eliminate transgene flow, a series of biological containment strategies have been developed, including transgene excision, chloroplast transformation, cytoplasmic male sterility and restorer genes, cleistogamy, etc. [2]–[11]. Although a steady progress has been achieved in recent decades, Hüsken et al. (2010) concluded that no single containment strategy would result in 100% reduction of gene flow, suggesting that combinations of complementary containment systems are required [12].

Generally, transgenic plants express full length genes encoding the active proteins of interest. Alternatively, target traits may be established by engineering functional proteins that result from reassembly of separately expressed inactive precursor peptides. The method, referred to as gene splitting, can be useful in controlling transgene flow.

The discovery of split inteins provides a useful tool for gene splitting studies. Inteins, referred to as “protein introns”, are internal protein elements that undergo self-splicing resulting in the ligation of flanking sequences (exteins) through a peptide bond to form a new mature protein [13]. Inteins were first discovered by two research groups in the yeast *Saccharomyces cerevisiae* [14], [15]. To date, more than 600 inteins have been described in organisms from all three domains of life [16]. Split inteins are capable of protein *trans*-splicing. An inactive target protein N-fragment (N-extein) fused to the N-terminal intein fragment (N-intein) and another inactive protein encompassing the C-terminal intein fragment (C-intein) fused to the target protein C-fragment (C-extein) could reassemble into a functional mature target protein, through intein mediated *trans*-splicing. *Ssp* DnaE, the first described split intein, was identified in the *Synechocystis* sp. strain PCC6803 [17]. *Ssp* DnaE is able to cyclize and trans-splice proteins in plants, such as tobacco [18], Arabidopsis [19] and wheat [20].

The *G_2_-aroA* gene (GenBank accession No.: EF155478) was identified from the G_2_ strain of *Pseudomonas fluorescens* isolated from glyphosate polluted area. This gene encodes the 445 aa EPSPS protein, which confers glyphosate resistance. The transgenic tobacco containing *G2-aroA* gene can tolerate 1.6% (the working concentration for control annual weed) isopropylamine salt of glyphosate (Roundup, 41.0% (W/V)) [21]. Dun et al. (2007) identified a suitable splitting site in *G_2_-aroA* named F295/T296. Using *Ssp* DnaE trans-splicing strategy, reassembly of the full-length and functional EPSPS protein in tobacco and *E. coli* was achieved [22]. In this study, we used *G_2_-aroA* as target gene and tobacco as model plant to assess the effect of gene splitting on transgene flow control. We confirmed the reconstitution of the functional EPSPS protein by *Ssp* DnaE intein mediated trans-splicing when the two gene split fragments were introduced into the same tobacco plant genome by genetic crossing. In addition, we demonstrated that successful reassembly was achieved in 100% cross hybrid plants. Furthermore, we found that gene splitting reduced the transgene flow by more than 75%. This is the first report on reassembly efficiency and effectiveness of transgene flow containment by gene splitting.

## Materials and Methods

### Genes and Germplasm

The *G_2_-aroA* gene encoding the glyphosate-resistant EPSPS protein was obtained from Lin lab of Biotechnology Research Institute of Chinese Academy of Agricultural Science. Pint-n (In) and Pint-c (Ic), the oligonucleotide sequences encoding the N-terminal (123 aa; Genebank accession no. AF545504) and C-terminal (36 aa; Genebank accession no. AF545505) domains of the *Ssp* DnaE intein, respectively, were kindly provided by Professor Thomas C. Evans, Jr. The vectors pImpactvector1.4 and pBinPLUS were purchased from Plant Research International (Netherlands). *E. coli* strains and other reagents were purchased from Takara company. Tobacco (*Nicotiana tabacum* NC89) seeds were conserved by our lab.

### Construction of expression vectors

DNAs encoding *EPSPS* segments, *Ic* and *In* were amplified with PCR using the following primers (restriction enzyme sites of *Nco*I (CCATGG), *Bgl*II (AGATCT) and *SacI* (GAGCTC) are underlined):

EPSPSn forward: 5′-GGCCCATGGATGGCGTGTTTGCCTGATGA-3′

reverse: 5′-GAAGTCCTGCGCGGCTACGC-3′

In forward: 5′–GCGCAGGACTTCAAATTTGCTGAATATTGCCT-3′

reverse: 5′–GGCAGATCTTTATTTAATTGTCCCAGCGTCAAG-3′

Ic forward: 5′–GGCCATGGATGGTTAAAGTTATCGGTCG-3′

reverse: 5′-GGATATGTTAAAGCAGTTAG-3′

EPSPSc forward: 5′-TTTAACATATCC-ACCCAGCCCGACGCCAAGGC-3′

reverse: 5′-CCGGAGCTCTCAGTCGTTTAGGTGAACGCCCAGG-3′

*EPSPSn-In* was then amplified with nested PCR using previous PCR products for *EPSPSn* and *In* as substrates, and EPSPSn forward and In reverse primers. The fusion gene *EPSPSn-In* was inserted into pImpactvector1.4 to generate the intermediate vector pIV1.4EnIn. The *EPSPSn-In* expression cassette was subcloned into the plant expression vector pBinPLUS to yield pBEPSPSn-In. Similar techniques were employed to construct the plant expression vectors pBIc-EPSPSc and pBG2-aroA.

### Transformation of tobacco

The three final plant expression vectors pBIc-EPSPSc, pBG2-aroA and pBEPSPSn-In were mobilized into *Agrobacterium tumefaciens* strain LBA4404 by the freeze-thaw method. Transformed bacteria were grown on YEB medium containing 100 mg/L kanamycin at 28°C and 150–250 rpm overnight. Cultures were diluted 1∶1 with YEB and allowed to grow to absorbance (measured at 550 nm) of ≈0.8. NC89 tobacco leaf discs from approximately 4-week-old shoot cultures were used for transformation with *A. tumefaciens*. After infection, leaf discs were incubated on a co-cultivation medium (1×MS salts, 3% sucrose, 2 mg/L 6-benzylaminopurine and 0.1 mg/L α-naphthalene acetic acid) at 28°C in the dark for 3–4 days and then selected on co-cultivation medium containing 500 mg/L cephalosporin and 100 mg/L kanamycin. The selected transgenic plantlets were then grown on media containing 1×MS salts, 3% sucrose, 100 mg/L kanamycin and 500 mg/L cephalosporin.

### Transgene insertion number analysis

T1 seeds of transgenic plants were germinated on a selective medium containing MS salt, 3% sucrose and 100 mg/L kanamycin. Pale and moribund seedlings were defined as kanamycin -susceptible (Kan^S^) plants, while healthy and green seedlings were considered kanamycin- resistant (Kan^R^). Numbers of Kan^R^ and Kan^S^ seedlings for each transformation event were analyzed by the ***χ***-squared test to identify plants with a single copy insertion. Furthermore, real-time quantitative PCR was used to assess copy number of the inserted *nptII* gene in transgenic plants. The primer pairs Ef (CTATCAGGACATAGCGTTGG)/Er (GCTCAGAAGAACTCGTCAAG) and Rf (GACGAAGCTTACTGAGGAAC)/Rr (CCAACAATCTATCAGCCACG) were designed according to gene sequences of *nptII* and *rnr2*, which encode neomycin phosphotransferase (the most frequently used marker in plant transformation experiments) and ribonucleotide reductase (endogenous reference), respectively. Real-time PCR was carried out individually with genomic DNA from single transgenic plants as templates on an AB 7500 Real Time PCR System (Applied Biosystems, USA) with the following reaction conditions: 30 sec at 95°C, followed by for 45 cycles of 5 sec at 95°C, 34 sec at 52.8°C, and 40 sec at 70°C. The initial *nptII* and *rnr2* template copy numbers were derived from CT values, and the inserted gene's copy number was estimated by the ratio of initial template copy number of *nptII* to that of *rnr2*.

### Selection of homozygous transgenic tobacco

Ten T1 seedlings were grown in soil, and T2 seeds were collected from individual plants and germinated on selective medium. The homozygous transgenic plants were recognized by healthy seedlings after three successive selections.

### Genetic crossing of tobacco plants

For cross-fertilization, pollen was collected from fully opened flower of homozygous male parent plants and dusted onto the stigma of homozygous female parent plants prepared from unopened buds.

### Western blot analysis

Soluble proteins were extracted from transgenic plant leaves using a Plant Protein Purification kit (Beijing CoWin Biotech Co., Ltd., China) according to manufacturer's instructions. Western blot detection of EPSPS peptides was carried following standard procedure with polyclonal antibodies raised in mice against EPSPSn-In and intact EPSPS (kindly provided by Lin's lab, Biotechnology Research Institute of Chinese Academy of Agricultural Science) at 1000 and 10000 dilutions, respectively.

### Evaluation of protein splicing reassembly efficiency

Hybrid seeds from genetic crossing plants were sterilized and inoculated onto medium containing MS salt, 3% sucrose and 33.8 mg/L glyphosate for germination. Seedlings with green leaves were considered glyphosate-resistant plant (gly^R^) and otherwise defined as glyphosate-susceptible (gly^S^). Seedlings were evaluated by leaf color (green or yellow) after 20 days of culture. Resistant and susceptible seedling amounts were analyzed by the ***χ***-squared test to assess the efficiency of protein splicing reassembly. In the leaf spraying experiment, 6 to 8-leaf-stage transgenic plants grown in the greenhouse were sprayed with 41.0% Roundup (isopropylamine salt of glyphosate as active ingredient) at indicated concentrations. The survival of the plants was evaluated after one week.

### Analysis of transgene insertion site

Tail-PCR was carried out to analyze the flanking sequences at the insertion sites of transgenic tobacco plants. A Genome Walking Kit (TaKaRa, Japan) was used to amplify the flanking sequences at target gene insertion sites. The specific primers F-1 (5′- GGACAGGTCGGTCTTGACAAAAAGAACCGG-3′), F-2 (5′- GTGCCCAGTCATAGCCGAATAGCCTCTCC-3′), F-3 (5′- CCTGCGTGCAATCCATCTTGTTCAATCATGCG-3′), and F-4 (5′- CGAGATAGGGTTGAGTGTTGTTCCAG -3′) were designed and synthesized based on the *nptII* gene sequence. Subsequently, three nested PCRs were carried out using primer pairs containing a specific primer and compound annex primers (AP1, AP2, AP3 and AP4) provided with the kit, with the genomic DNA as template. The obtained sequences were analyzed by comparison with the GenBank and tobacco genome databases.

### Evaluation of the effect of gene splitting on transgene flow control

Hybrids harboring both *EPSPSn-In* and *Ic-EPSPSc* were selfed and artificially back-crossed to the non-transgenic tobacco line NC89. The resulting seeds were sterilized and inoculated onto media containing MS salt, 3% sucrose and 33.8 mg/L glyphosate to assess glyphosate resistance of the hybrid progeny. The gly^R^/gly^S^ segregation ratio was calculated, and statistical analyses were carried out using the chi squared test.

## Results

### Fusion between EPSPS and Ssp DnaE intein

According to the suitable and effective splitting site (F295/T296) reported by Dun et al. [22], divided *G_2_-aroA* gene segments were fused to *Ssp DnaE* segments. The resulting constructs encoded two fusion proteins, EPSPSn-In and Ic-EPSPSc. EPSPSn-In is an in-frame fusion between the first 295 amino acid residues of the EPSPS protein and the 123 amino acid residues of *Ssp* DnaE intein-N. Likewise, Ic-EPSPSc is an in-frame fusion between the 36 amino acid moieties of Ssp DnaE intein-C and the remaining 150 EPSPS amino acid residues.

**Figure 1:**
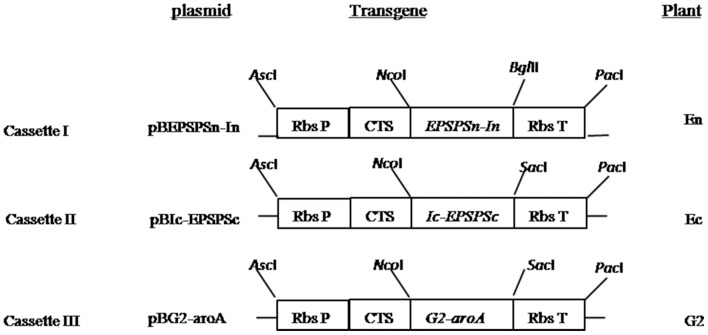
Summary of transgene constructs used for tobacco transformation. Construct names, gene expression cassettes, and names of transgenic plants are presented. Rbs P represents the promoter of *chrysanthemum* Rubisco small unit gene (*RbcS*); CTS is the chloroplast signal peptide containing a natural intron from the *RbcS* gene of *chrysanthemum*; and Rbs T represents the terminator of *chrysanthemum RbcS*.

The fused genes were cloned into ImpactVector1.4 vector. The vector contains a Rubisco small subunit promoter from which the target genes were transcribed, and a Rubisco small subunit terminator (RbcS1 T) from *Asteraceous chrysanthemum* at the 3′ end[23]. The vector also contains a signal peptide of the first 11 amino acids from *Chrysanthemum morifolium* Rubisco small subunit protein fused at the N terminus to deliver target proteins into chloroplast stroma[23]. The expression cassettes were then subcloned into T-regions of plant expression vector pBinPLUS. This resulted in three expression plasmids: pBEPSPSn-In, pBIc-EPSPSc and pBG2-aroA. The T-regions in all plasmids harbored nosP::nptII::nosT of kanamycin resistance marker. The structures of the three plasmids are summarized in Fig. 1.

### In vivo reassembly efficiency of the split EPSPS through intein mediated trans-splicing

Infection with pBEPSPSn-In, pBIc-EPSPSc or pBG2-aroA plasmids resulted in three sets of transformed plant lines, labeled as En, Ec and G2, respectively. Totally 35 of En, 42 of Ec and 60 of G2 transgenic tobacco lines were obtained by agrobacterium mediated transformation. T1 seeds of transgenic tobacco were germinated on 100 mg/L kanamycin selective medium. Analysis of segregation ratio showed that the seeds from plant lines En-33, Ec-11 and G2-24 exhibited Kan^R^: Kan^S^ seedling at 3:1 ratio, as would be predicted from single copy transgenic insertions.

In order to confirm the insertion number of transformed genes, real-time PCR was used to detect the copy numbers of the exogenous *nptII* gene in En-33, Ec-11 and G2-24. The standard curve of the reference gene *rnr2* was CT  = 33.953–3.069log(cn), with r^2^ and amplification efficiency of 0.998 and 111.752%, respectively, where CT is the cycle threshold and cn is the copy number. The standard curve of *nptII* was CT  = 35.236–3.313log(cn), with r^2^ and amplification efficiency of 0.999 and 100.382%, respectively. The *rnr2* and *nptII* copy number (cn) values were derived from the standard curves and CT values obtained in real-time PCR. The cn ratios of *rnr2* to *nptII* for En-33, Ec-11 and G2-24 were 1.06, 0.95, and 1.34, respectively, indicating a single copy insertion of *nptII* in the transgenic tobacco lines En-33, Ec-11 and G2-24.

**Figure 2:**
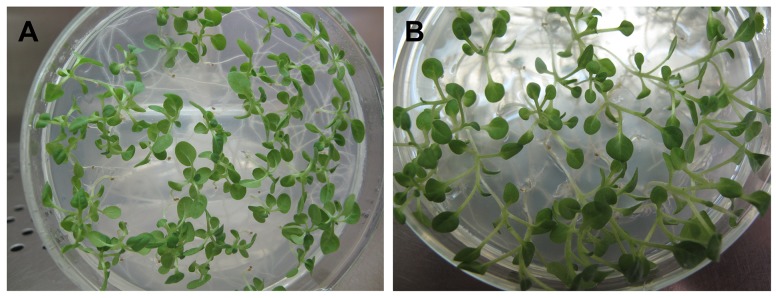
Growth of homozygous transgenic plant lines homEn-33 (A) and homEc-11 (B). T3 seeds from transformed plants were germinated on selective media containing MS salt, 3% sucrose and 100 mg/L kanamycin. Pale and moribund seedlings represented kanamycin-susceptible (Kan^S^) plants, while healthy and green seedlings were kanamycin- resistant (Kan^R^). All homEn-33-5 and homEc-11 seedlings grew healthy and displayed kanamycin resistance.

T0 plantlets of En-33 and Ec-11 were planted in a greenhouse. The seeds were collected at harvest and germinated on MS0 medium containing 100 mg/L kanamycin. The kanamycin resistant aseptic seedlings were then again transferred to greenhouse culture. After three rounds of selfing, homozygous lines of En-33 and Ec-11 were obtained and named homEN-33 and homEC-11, respectively. All seeds of homEn-33 and homEc-11 seedlings grew healthy on kanamycin selective medium (Fig. 2). For genetic crossing, homEn-33 was used as pollen donor, whereas homEc-11 was pollen recipient. The resulting hybrids were designed En-33×Ec-11.

**Figure 3:**
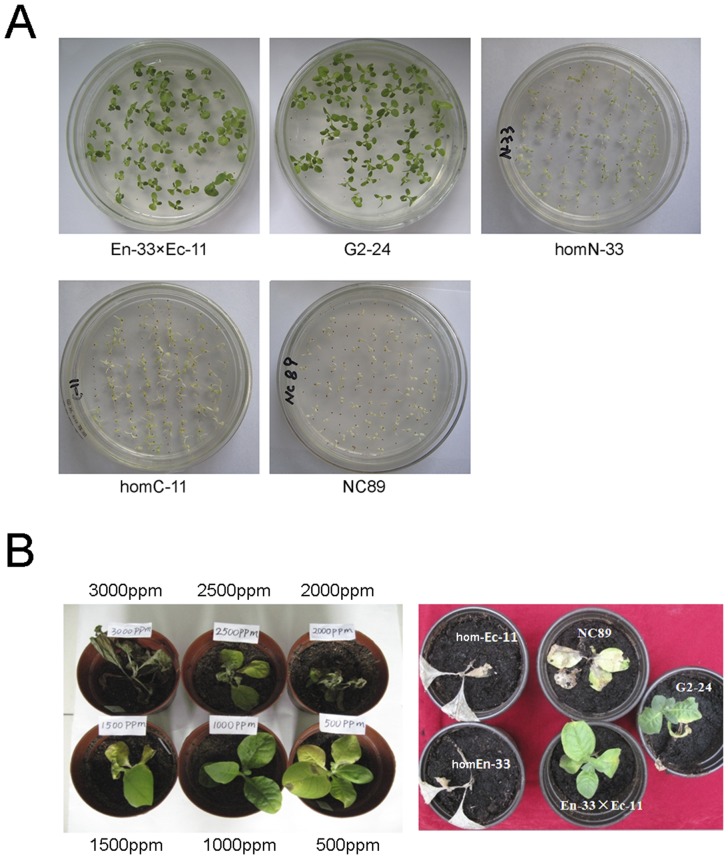
Analysis of glyphosate resistance in the transgenic tobacco plants. A. Growth of different transgenic tobacco lines on glyphosate selective medium. Seeds were sterilized and inoculated onto medium containing MS salt, 3% sucrose and 33.8 mg/L glyphosate for germination. The seedlings with green leaves represented glyphosate-resistant plants and otherwise defined as glyphosate-susceptible. B. Grown plants (6 to 8-leaf stage) were tested for glyphosate resistance. Left: NC89 plants one week after spraying different concentrations of 41% Roundup herbicide. Right: transgenic tobacco plants one week after spraying 2500 ppm Roundup.

**Figure 4:**
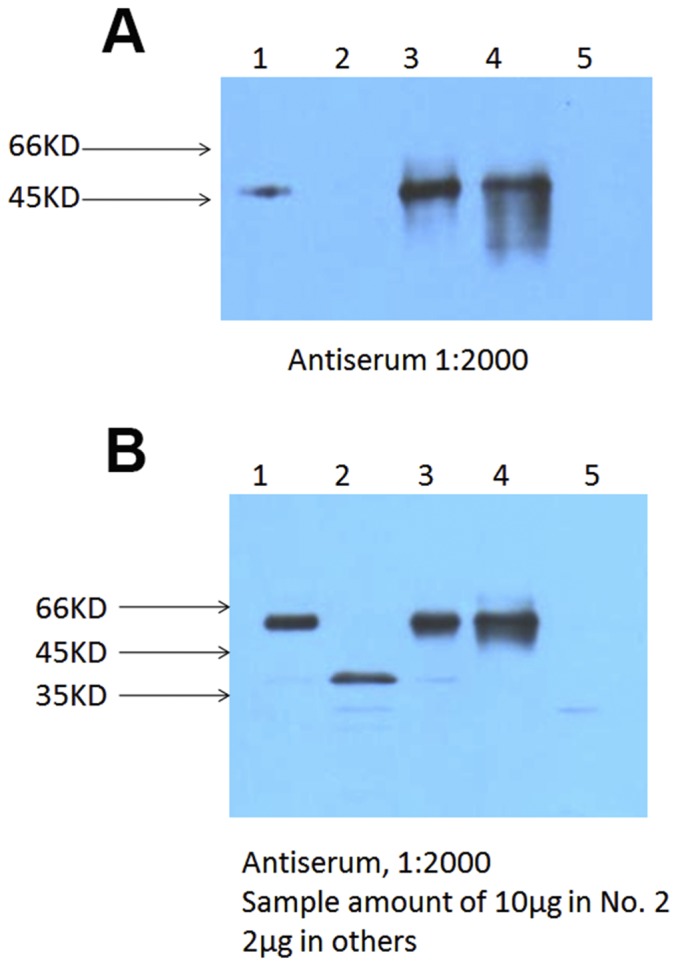
Western blot analysis of EPSPS peptides. Proteins from different plants were blotted with antibodies against EPSPSn-In (A) or full length EPSPS (B).1: En-33; 2: Ec-11; 3: G2-24; 4: En-33×Ec-11; 5: NC89.

To assess the efficiency of protein reassembly through intein mediated trans-splicing, glyphosate resistance of hybrid En-33×Ec-11 was analyzed by seed germination on selective medium containing 33.8 mg/L glyphosate. As controls, seeds of hybridization parent homEn-33, homEc-11 as well as G2-24 and non-transgenic NC89 were germinated on selective and nonselective media. All En-33×Ec-11 hybrid seedlings grew normally on glyphosate selective medium and displayed similar glyphosate resistance phenotype compared to G2-24, which contained the full length of the glyphosate resistance gene (Fig. 3A). In addition, glyphosate resistance was tested by leaf spraying experiment in 6 to 8-leaf-stage transgenic plants. Plants grown in the greenhouse were sprayed with the 41.0% Roundup (isopropylamine salt of glyphosate as active ingredient) at doses of 2500 ppm. Within one week, the homEn-33, homEc-11 and NC89 plants wilted and turned yellow, gradually dying, whereas the hybrid En-33×Ec-11 and G2-24 transgenic tobacco plants grew normally (Fig. 3B). These results indicated the successful reassembly of a functional EPSPS protein from EPSPSn-In and Ic-EPSPSc by intein mediated protein trans-splicing. These findings were further confirmed by Western blot assays. As shown in Fig. 4, in the hybrid En-33×Ec-11 plants, accumulation of the reassembled full length EPSPS protein with slight larger size than EPSPSn-In was observed. Meanwhile no smaller Ic-EPSPSc was detected in hybrid plants, indicating a highly efficient trans-splicing induced reassembly.

### Gene splitting and control of transgene flow

**Figure 5:**
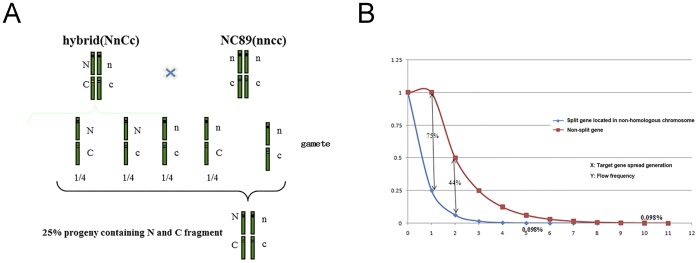
Gene flow frequency with gene splitting strategy. A. Prediction of glyphosate resistant plants percentage in the En-33×Ec-11 backcrossing progeny. N represents the EPSPSn-In fragment; C stands for the Ic-EPSPSc fragment. According to the Mendel's laws of inheritance, if the two genes were inserted into different chromosomes, 25% backcrossing progeny would contain both genes and display glyphosate resistance. B. Percentages of progeny plants resulting from backcrossing with gene splitting or full length transgenic strategy were compared. At F1 generation, 25% of hybrid backcrossing progeny would display the target character, 75% lower than backcrossing progeny plants with full length transgenic and wild type plants. After 5 generations, the ratio of backcrossing transgenic plants using gene splitting strategy would display the target character in less than 0.1% population.

The analysis of En-33 and Ec-11 flanking sequences by thermal asymmetric interlaced–PCR showed that the two transgenic inserts were located in different chromosomes. According to Mendel's laws of inheritance, without possibility of chromosome crossing-over, 25% pollen possess A and B genes at the same time if A and B were located at different chromosomes in a given plant (Fig. 5A). Upon the backcrossing between En-33×Ec-11 hybrid (as pollen donor) and non-transgenic tobacco NC89 (as pollen recipient), 664 of 2812 back-crossed progeny plantlets displayed glyphosate-resistance, a gly^R^ ratio of 23.61%. While self-crossed, 2328 of 4158 progeny seeds of En-33×Ec-11 germinated on glyphosate selective medium were glyphosate-resistant, a ratio of 55.99%. As estimated by the ***χ***-squared test, both data fit the hypothesis that *EPSPSn-In* and *Ic-EPSPSc* were inserted into different chromosomes according to Mendel's laws of inheritance. After five generations, lower than 0.1% progeny plants resulting from backcrossing between hybrid and wild type would be expected to contain both splitted gene fragments for the reassembly of full length functional protein (Fig. 5B). The same threshold decrease would be expected after 10 generations if the transgene was carried with full length target gene. Therefore gene splitting technique would significantly reduce transgene flow. At F1 generation, the reduction would be expected to be at least 75%.

## Discussion

Transgene flow continues to pose a threat on environment and food safety, and has therefore become a major concern with increasing production of genetically modified organisms [27]-[29]. Intein mediated protein trans-splicing may limit the environmental impact of a foreign gene by keeping different parts in different chromosomes while assembling gene products in one cell through crossing to achieve the desired function [18], [24], [25].

Ssp DnaE intein has been used by others to cyclize and trans-splice proteins in various plants, such as tobacco, Arabidopsis and wheat [18]–[20]. It was recently proposed that gene flow from such transgenic plants to wild or weedy relatives would transmit only a portion of the full-length gene, which should imply reduced environmental impact [18], [24], [25]. However, to optimally apply the gene splitting technique for containment of transgene flow, it is critical to ensure reassembly efficiency. Few reports on detection and analysis of reassembly efficiency are available. Iwai et al. (2006) estimated the efficiency of ligation by trans-splicing using band intensities after sodium dodecyl sulfate–polyacrylamide gel electrophoresis [26]: the reassembly efficiency was determined by comparing the molar amount of the ligated product and either of the residual N- or C-terminal precursor fragments, a useful method for prokaryotic expression systems. However, this approach is not suitable for plant expression systems due to the laborious purification of target proteins. Therefore, it is critical to design new methods to estimate reassembly efficiency after gene splitting. In this study, based on the phenotype of glyphosate-resistant hybrids, the reassembly efficiency of the target protein was estimated at the plant level. Our data indicated that 100% En-23×Ec-11 hybrid plants tolerate glyphosate treatment, suggesting perfect functional reassembly efficiency after gene splitting. This is the first report on reassembly efficiency after gene splitting in a plant expression system.

Transgenic plants containing divided target gene incorporated into different chromosomes are with less risk to pass the transgenic products into environment. Our results showed that less than 25% of progeny plants still expressed reassembled functional EPSPS proteins when the hybrid transgenic plants were backcrossed to wild type species. Indeed, the gene splitting technique allows the two gene segments to be located on different chromosomes instead of expressing the full length gene on a single chromosome; according to Mendel's laws of inheritance, the latter situation would result in 100% inheritance in the first hybrid generation, while only 25% should be expected with gene splitting. Importantly, the percentage kept decreasing after passing to more generations.

Overall, our results demonstrate that the gene splitting technique can effectively reduce transgene flow, providing a new biological containment strategy in the biosafety field. It is worth mentioning that a series of biological strategies for transgene flow containment have been devised, each with unique characteristics and suitable application scale. It is difficult to control transgene flow completely using only one strategy. Therefore, future studies should focus on controlling transgene flow by combining two or more strategies.
